# COVID-19 Surveiller: toward a robust and effective pandemic surveillance system based on social media mining

**DOI:** 10.1098/rsta.2021.0125

**Published:** 2022-01-10

**Authors:** Jyun-Yu Jiang, Yichao Zhou, Xiusi Chen, Yan-Ru Jhou, Liqi Zhao, Sabrina Liu, Po-Chun Yang, Jule Ahmar, Wei Wang

**Affiliations:** Department of Computer Science, University of California, Los Angeles, CA 90024, USA

**Keywords:** pandemic surveillance, social media mining, knowledge graph, natural language processing

## Abstract

The outbreak of the novel coronavirus, COVID-19, has become one of the most severe pandemics in human history. In this paper, we propose to leverage social media users as social sensors to simultaneously predict the pandemic trends and suggest potential risk factors for public health experts to understand spread situations and recommend proper interventions. More precisely, we develop novel deep learning models to recognize important entities and their relations over time, thereby establishing dynamic heterogeneous graphs to describe the observations of social media users. A dynamic graph neural network model can then forecast the trends (e.g. newly diagnosed cases and death rates) and identify high-risk events from social media. Based on the proposed computational method, we also develop a web-based system for domain experts without any computer science background to easily interact with. We conduct extensive experiments on large-scale datasets of COVID-19 related tweets provided by Twitter, which show that our method can precisely predict the new cases and death rates. We also demonstrate the robustness of our web-based pandemic surveillance system and its ability to retrieve essential knowledge and derive accurate predictions across a variety of circumstances. Our system is also available at http://scaiweb.cs.ucla.edu/covidsurveiller/.

This article is part of the theme issue ‘Data science approachs to infectious disease surveillance’.

## 1. Introduction

COVID-19, which is one of the most fatal pandemics in human history, has already changed our lives and resulted in substantial and lasting impacts in many domains, such as public health, economy and society. As of May 2021, COVID-19 has globally infected more than 160 million people with over 3 million deaths [1]. To alleviate the damage from COVID-19 and potential epidemics in the future, it is urgent to establish an effective and robust surveillance system to automatically and precisely monitor the spread of pandemics and estimate the risk factors across different areas. For example, the government can allocate medical resources earlier and provide more health education for residents if the system predicts the increments of further infections. Public health researchers can have better attribute models if the system can precisely suggest the risk factors for an area, such as crowded events or inappropriate behaviours.

To predict the pandemic spreads, conventional methods usually utilize epidemiological models, such as the susceptible, infected and recovered (SIR) model [2], the susceptible, infectious, susceptible (SIS) model [3] and the herd immunity threshold [4]. However, these traditional methods suffer from using only homogeneous historical case numbers for deriving prediction models while the spreads of moderns diseases are usually more complex and related to many real-world events. Without observing and sensing real-world events, predictive models based on historical records are incapable of monitoring the pandemic spreads which could be potentially far from previous numbers. Moreover, these models only estimate numerical predictions over time, such as infection cases and deaths. In other words, they usually succumb to provide meaningful insights for public health researchers and domain experts to make governmental and clinical decisions. Although the government can always hire people to collect the real-world events related to the epidemics, it is highly expensive to manually collect an enormous amount of information on a long-term basis while manual surveys are usually accompanied by significant delays. Hence, constructing an approach to automatically collect relevant knowledge becomes one of the biggest challenges to establish robust and effective pandemic surveillance systems.

To automatically capture real-time dynamics, social media can be considered as a great platform to provide sufficient knowledge while we treat social media users as ‘social sensors’ [5] to detect real-world events. For example, Twitter users can reflect the air quality [6] and earthquakes [7] in the surrounding areas. However, pandemics are much more complex than those natural events so that analysing individual tweets can be insufficient to provide enough evidence for monitoring pandemic spreads and suggesting risk factors.

The other considerable challenge of establishing effective and robust pandemic surveillance systems is the interaction between humans and machines. Although machine learning models are capable of providing accurate predictions, these models are usually trained and deployed on computational servers with input and output data in unreadable formats. Therefore, there is a gap between these computational models and end users like public health researchers without computer science background. As a result, to maximize the impacts of pandemic surveillance models, it is essential to build a user-friendly interface for not only processing input parameters but also conveniently visualizing the results.

In this paper, we propose the COVID-19 Surveiller to address the above challenges for establishing a robust and effective pandemic surveillance system for COVID-19 based on deep learning and full-stack system development. More specifically, the framework consists of three parts, including the tweet crawler, social media mining for pandemic surveillance and full-stack system development. For the tweet crawler, we collaborate with Twitter to use their COVID-19 streaming API to collect large-scale tweets that contain COVID-19 related keywords. For pandemic surveillance, we first construct a temporal heterogeneous knowledge graph by named entity recognition (NER) and relation extraction. A well-designed dynamic graph neural network is then applied to appropriately model the temporal dynamic for forecasting pandemic trends and suggesting risk factors. We also developed an interactive and intelligent web system to demonstrate COVID-19 Surveiller. To show the effectiveness of COVID-19 Surveiller, the extensive experiments show the significant improvements of our approach over conventional methods. We also conduct in-depth case studies to indicate the convenience of COVID-19 Surveiller for end users.

## 2. Related work

### (a) Compartment models

Originally proposed by Ross [8], the compartment models express the dynamics of infectious diseases using ordinary differential equations (ODEs). One of the simplest and most prevailing compartment models is the **SIR** model [9–12]. In their framework, the population is segmented into one of several compartments, e.g. **S**usceptible, **I**nfectious or **R**ecovered. A set of evolving equations are accompanied to express the population flow among these population categories. The model is intrinsically dynamic in that the numbers in each compartment may fluctuate over time. Based on **SIR**, many derivatives are developed to complement this line of research. For example, the **SIS** model [2] considers the diseases that do not confer any long-lasting immunity, so that the recovered population can become infected again. The **SIRD** model [13] differentiates between **R**ecovered individuals and **D**eceased. The **MSIR** model [14] takes passive immunity into account covering several diseases such as measles. To explicitly model the carrier state where some people might have been infected while not suffering the symptoms, the **SEIR** [15] incorporates the **E**xposed compartment. Similarly, the **SEIS** [16], **MSEIR** [17] and **MSEIRS** extend the **SEIR** by taking into account no immunity, passive immunity and temporary immunity. Since the outbreak of COVID-19, due to its huge impact on daily life and economic activities, massive research based on compartment models has been carried out to specifically focus on COVID-19 risk factor modelling. Since there could be an incubation period for those people infected with COVID-19, the number of reported cases might not reflect actual numbers as many infectious cases have not been tested. **SuEIR** [18] extends **SEIR** in the way that it explicitly models the untested/unreported compartment.

### (b) Time-series model

Time series forecasting is a task that has drawn attention for a long time. Conventional methods, such as autoregressive integrated moving average (ARIMA), usually make the assumption that the future time series have linear relationships with the past ones. Qin *et al.* [19] proposed a dual attention mechanism that can adaptively select the most relevant input features and capture the long-term temporal dependencies of a time series. However, the memory capability of LSTMs is still limited [20]. To tackle the intrinsic problem of LSTM, some works proposed to create an external memory to explicitly store some representative patterns that can be frequently observed in the history [21]. Transformer [22] is another solution to vanishing memory that only consists of an attention component. Overall, Wavenet [23] and TCN [24] are the state-of-the-art models for any kinds of time series forecasting. Specifically, there has been quite a lot of works dealing with COVID-19 related forecasting. Le *et al.* [25] combine recurrent neural networks with an autoregressive model and train the joint model with a specific regularization scheme that increases the coupling between regions. Rodrıguez *et al*. [26] opt to use a feedforward network with autoregressive inputs to incorporate short-term dependencies in the time series. Jin *et al.* [27] developed an attention-based method that makes forecasts via comparing patterns across time series obtained from multiple regions. Released by Facebook, Prophet [28] is an additive model that emphasizes seasonal effects, so that a time series that changes periodically works better on that model.

### (c) COVID-19 surveillance systems

A variety of efforts have been put into developing COVID-19 surveillance systems. These systems aims at giving people and policy makers a better sense of how severe the pandemic is, and help them make better decisions to reduce the spread of the virus. They usually collect historical COVID-19-related stats, and visualize them in ways such as coloured map and curve charts to intuitively demonstrate information on accumulative cases in every county and their trends. Representatives of these systems include JHU Coronavirus Resource Center,^1^ Worldometers.info^2^ and 1point3acres.^3^

Although extensive existing studies have been carried out to address time series prediction, the majority of them only leverage historical time series observations as the only input. When it comes to COVID-19 risk factor forecasting, only looking at historical risk factors such as case numbers and death numbers might be insufficient to make a precise forecasting. A key insight is that some social events, such as the LA marathon and racial equality parades, can seriously impact the case and death numbers. Moreover, unearthing the relationships between social events and risk factors can help public health experts to make better plans to reduce the risks by suggesting political policies to government personnel. As a result, we can benefit from both the precision and interpretability perspectives by taking social events into account.

## 3. Methods

### (a) COVID-19 Surveiller: framework overview

**Figure 1.**
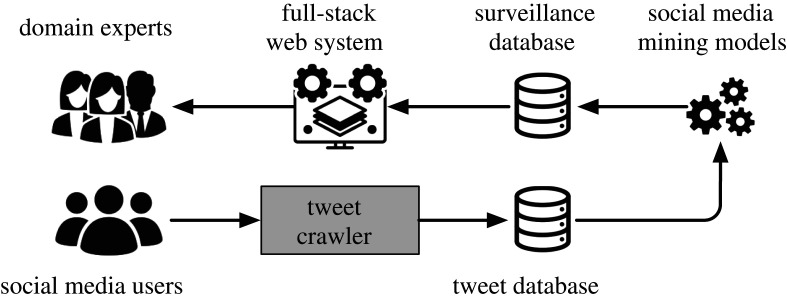
Illustration of our proposed framework for pandemic surveillance, COVID-19 Surveiller.

As shown in figure 1, in this paper, we propose COVID-19 Surveiller to monitor the pandemic trend and suggest risk factors across different locations. We first collect disease-related tweets from social media users based on a tweet crawler and index them in a tweet database. Based on collected tweets over time, we establish social media mining models to treat social media users as social sensors [6,7], thereby forecasting the information about the pandemics (e.g. infection cases and deaths) and inferring a list of risk factors, such as hazardous real-world events. To facilitate the user experience, we build a user-friendly full-stack web system so that users can interact with the system to conveniently monitor the pandemics by querying the prediction times and locations.

#### (i) Tweet crawler

To steadily obtain social media tweets, in our work, we collaborate with Twitter and implement a real-time tweet crawler using their COVID-19 streaming API.^4^ Specifically, the streaming API returns comprehensive and tweets related to COVID-19 based on Twitter’s internal COVID-19 tweet annotation on a real-time basis. As a result, the full disease-related conversations on Twitter can provide a strong foundation for pandemic surveillance.

#### (ii) Robust full-stack web system development

We describe the detailed design of our full-stack web system in the electronic supplementary material, section S1. We also conduct several case studies for the use cases of the web interface as shown in electronic supplementary material, section S2.

### (b) Social media mining for pandemic surveillance

#### (i) Constructing activity nodes from free-text data

**Figure 2.**
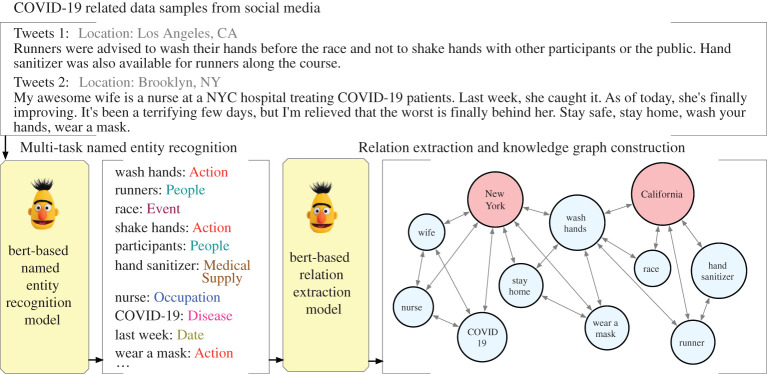
The pipeline of constructing activity nodes from free-text data. (Online version in colour.)

To recognize potential risk factors in the rich collection of COVID-19 social media data, we propose a bottom-up approach. As shown in figure 2, we employ NER to extract entities of interest from the social media data and apply the relation extraction (RE) to identify potential relationships among the entities. We also pre-train a deep language model to provide domain-specific contextual representations. We then incorporate the extracted entities and relationships to build a heterogeneous knowledge graph.

#### (ii) Named entity recognition

NER has been widely studied in the natural language processing fields, but most traditional NER approaches require heavy feature engineering including parsing the Part-of-Speech tags of each word and syntactic dependency structures of each sentence [29–31]. Some recent works incorporate neural networks to improve the extraction performance. The authors in [32–34] ensemble conditional random fields [35] with convolutional neural networks [36] or recurrent neural networks [37], requiring extensive human annotation effort at the training stage which is expensive and time-consuming. We thus collect datasets from multiple tasks including I2B2-2010 [38], CORD-NER [39] and MACCROBAT2018 [40] and jointly fine-tune a deep language model to encode the tokens from the social media data. One layer of the Feed Forward Network (FNN) [41] with softmax [42] takes the hidden representations of each token as input and outputs the category of this token. For example, we aim to classify the *hand wash* as an *Action* while recognize *race* as an *Event*. Without loss of generality, we make use of the BERT model [43] to build the NER model. ELMo [44] or RoBERTa [45] can also be applied.

#### (iii) Relation extraction

We then extract relations among the recognized entities. Previous methods [46–49] rely heavily on the quantity and quality of the annotated datasets to achieve satisfactory performance on predicting the relation type. Therefore, these methods are not suitable for identifying emerging relation types. As a result, we simplify the task into a binary classification problem, i.e. determining whether a relation exists between two recognized entities. We aggregate datasets from multiple tasks including Wiki80 [50], I2B2-2012 [51] and MACCROBAT2018 [40] to generate the positive instances, i.e. sentences containing two entities and a *True* relation between them. We also conduct negative sampling to create instances with label *False*. In order to build a balanced binary dataset, we take the same amount of negative samples as the positive ones. We ultimately fine-tune another contextualized language model based on BERT to learn the sentence representations. A binary classifier comes after to conduct the binary classification.

#### (iv) Pre-trained language model

Inspired from a few recent work on pre-training the language models [52–54] with domain-specific corpus for tasks like bioinformatics knowledge acquisition and clinical information extraction [55,56], we obtain the large tweet dataset and all the COVID-19 relevant text corpus and pre-train a CoronaBERT model with 12 layers of transformers [57] and 110 M parameters. This pre-trained language model provides domain-specific token and sentence representations. We continuously update the CoronaBERT as more COVID-19 data become available and will release it on a quarterly basis to facilitate the research community.

#### (v) Knowledge graph aggregation

After extracting the entities and relations, it is straightforward to aggregate them into a knowledge graph $G^{\left(t\right)}=(V^{t},E^{\left(t\right)})$ at time t where the node set $V^{\left(t\right)}$ includes location nodes $V_{L}^{\left(t\right)}$ and entity nodes $V_{E}^{\left(t\right)}$. The edge set $E^{\left(t\right)}$ is composed of three types of edges: *Location–Location* edges, *Location–Entity* edges, *Entity–Entity* edges. We build a *Location–Location* edge between two location nodes if they are neighbouring to each other in the US map or we find population transition from the mobility dataset. We obtain the mobility dataset from SafeGraph.^5^ We build a *Location–Entity* edge between a Location node and an Entity node if the entity is extracted from a tweet that was posted in that location. We also construct an *Entity–Entity* edge between two entity nodes if a *True* relation is identified between them.

#### (vi) Dynamic graph neural network for monitoring pandemics and risk factors

Given a sequence of knowledge graphs $G^{\left(1\right)},G^{\left(2\right)},\ldots ,G^{\left(T\right)}$ built on the extracted entities and relations, we aim to learn rich node representations over time by encoding both temporal evolution patterns and structural neighbourhood information [58], which can be useful for monitoring the pandemics and identifying the risk factors. Specifically, we formulate the task into a time-series prediction problem. With the knowledge graphs and historical statistics of COVID-19 confirmed case and fatality, we predict the case and fatality numbers in the short-term and long-term future. Simultaneously, we detect the events of high risks leading to emerging cases or deaths over different locations and times. Traditional machine learning models [11,12,28,59] either segment COVID-19 populations into susceptible, infectious or recovered groups, or detect the trend, seasonality and holiday patterns. However, these approaches can neither incorporate multi-dimensional textual features nor leverage the graph structures for propagating information to neighbouring nodes. To overcome the above challenges, we propose a dynamic graph neural network (DGNN) architecture that employs the Graph Attention Network (GAT) to encode the knowledge graphs and a Bidirectional Recurrent Neural Network (BiRNN) with Gated Recurrent Unit (GRU) to encode the sequential patterns for confirmed case and fatality prediction. Besides this, we employ the attention scores computed in the GAT module to retrieve the location-wise risk factors for the increasing trends of COVID-19.

**Figure 3.**
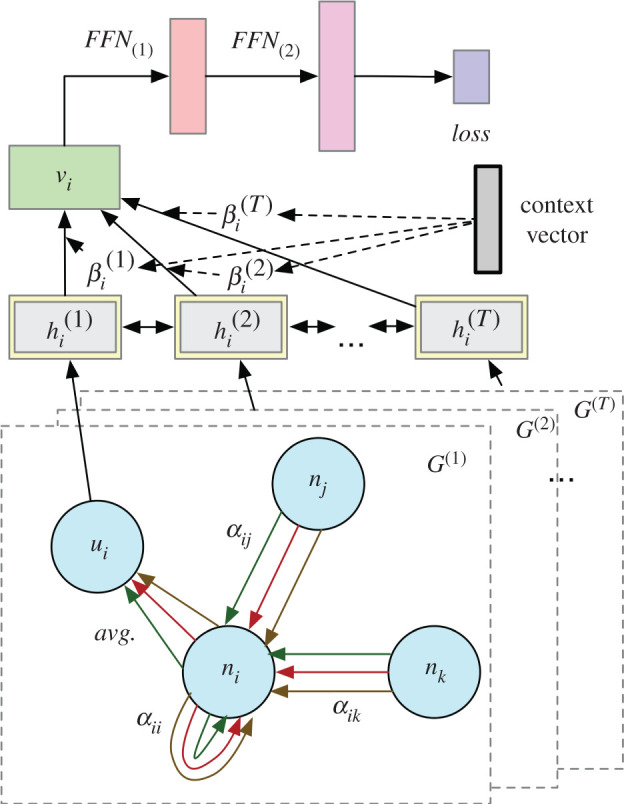
Time series prediction model. (Online version in colour.)

As shown in figure 3, we first leverage a multi-head graph attention model to pass the message from the neighbours to each node to encode the contextual knowledge:

$$u_{i}=\frac{1}{H}\sum_{p=1}^{H}\sigma \left(\sum_{j\in N(i)}\alpha_{ij,p}z_{i,p}\right),\;z_{i,p}={\text{W}}_{p}n_{i}$$

and

$$\alpha_{ij,p}=\frac{\text{exp}(s_{ij,p})}{\Sigma_{j^{\prime }\in N(i)}\text{exp}(s_{ij^{\prime },p})},\;s_{ij,p}=\text{LeakyReLU}(w_{p}^{T}(z_{i,p}||z_{j,p}))$$

where p denotes the index of attention head; N(i) indicates the neighbours of $n_{i}$ in the graph; $W_{p}$ is a weight matrix for feature projection under head p while $w_{p}$ is a weight vector; σ(⋅) and LeakyReLU [60] are nonlinear activation functions; exp is the exponential function. $s_{ij,p}$ represents the importance score of the edge between nodes i and j. We finally compute an average representation $u_{i}$ over all the heads.

In order to take advantage of the temporal dependencies among the same location node i of different times, we feed a sequence of $u_{i}$ from T graphs to a BiRNN to learn a latent representation. Here, we choose Gated Recurrent Unit (GRU) [61] instead of Long-short Term Memory unit (LSTM) [62] due to its simple structure and computational efficiency [63]. Then we apply the attention mechanism to compute weights $\beta_{i}^{\left(t\right)}$ for aggregating the hidden representations of different times to $v_{i}$. Two layers of FFN with nonlinear transformations convert $v_{i}$ to a scalar, representing the predicted case or fatality ${\hat{y}}_{i}^{T+r}$ for location i at day T+r. r is a variable, denoting the number of days ahead to predict. We choose the mean squared error as our loss function:

$$L=\frac{1}{mn}\sum_{t=1}^{n}\sum_{i=1}^{m}(y_{i}^{\left(T+r\right)}-{\hat{y}}_{i}^{\left(T+r\right)}{)}^{2},$$

where $y_{i}^{\left(T+r\right)}$ denotes the ground truth of the confirmed case/fatality for location i at day T+r. m and n are the number of locations and data points, respectively.

## 4. Results

In this section, we describe the details about our system environments and showcase some experimental results. The results demonstrate that not only the integrated forecasting algorithm is effective, the design and user interface is neat and user-friendly. Specifically, we conduct extensive experiments for quantitative analysis and several case studies to show how our system facilitates the pandemic surveillance.

### (a) System settings

Our demo system is available at http://scaiweb.cs.ucla.edu/covidsurveiller/. We further introduce more details about our system settings as follows.

#### (i) Dataset statistics

For social media mining, we have collected 270 k tweets published in the USA from the Twitter COVID-19 streaming API every day, starting from 15 May 2020. For the statistics of pandemic trends, the average numbers of new confirmed cases and fatalities over all states in the USA are 1788 and 29 with the standard deviations of 3374 and 63.

#### (ii) System environments

For the full-stack web system, we deploy the system on a Linux-based computational server with 512 GB memory and 148 TB storage on a fast network file system shared with the machine learning server. We train our social media mining models on a machine learning server with 512 GB memory, an Nvidia V100 GPU and the shared storage.

#### (iii) Model implementation

We train the NER and RE models for a maximum of 10 epochs while learning the time series prediction model for at most 300 epochs. All the models are implemented in PyTorch and the Adam [64] is used for optimizing the parameters. We apply early stopping in the training phase to avoid over-fitting. We also use dropout and batch normalization to the outputs of DGNN and GGN layers to avoid the over-fitting. In the pre-processing step, we filter out the tweets that contain more than 40 tokens (0.17%) to keep the GPU computation efficient. We also focus on the English tweets by removing the tweets containing 90% non-English tokens. We use one Nvidia V100 GPU to train the models and all the experiments can be finished within 10 h. We apply grid search to find the optimal hyperparameters. After the grid search, we set dropout rate, batch size, learning rate, graph sequence length T as 0.5, 4, 0.001 and 7, respectively.

### (b) Comparative baselines

To demonstrate the significance of our proposed approach, we compare with three categories of baseline models, including compartment models, statistical models and neural network-based methods.

— Compartment models
  1. RobertWalraven-ESG [65] approximates the SEIR model with a mathematical model that initialized from a particular skewed Gaussian distribution.
  2. UCLA-SuEIR [18] further considers Untested/Unreported compartment than the SEIR model based on the fact that exposure to the virus can also infect the susceptible group in a certain period.
  3. JHU_IDD-CovidSP [66] is a variant of the SEIR model which aims to generate more realistic infectious periods by employing an Erlang distribution to model the time in the Infected compartment.
— Statistical models
  1. ARIMA [67] is an autoregressive moving average model and leverages the past values to explain the given time series.
  2. PROPHET [68] pays more attention to the nonlinear trends of seasonality and holiday effects to make time series prediction.
— Neural network-based methods
  1. LSTM [69] uses a Recurrent Neural Network with two layers of LSTM to learn the temporal dependencies in the time series prediction.
  2. MPNN and MPNN+LSTM [70] are message passing neural network-based models [71] and aggregate the past values in a location mobility graph. MPNN+LSTM combines MPNN and LSTM to jointly model the message passing and temporal dependencies.

### (c) Evaluation of pandemic surveillance

As the backbone of COVID-19 Surveiller, social media mining models play an important role in pandemic surveillance. Hence, we first evaluate the performance of our pandemic surveillance model proposed in §3(b).

#### (i) Named entity recognition and relation extraction

To verify the effectiveness of the NER module, we remove all the entity nodes from the knowledge graph and only use the location nodes to predict the confirmed case number. The result shows that there is an 8.9% greater error without considering entity nodes. For the RE module, we remove all the *Entity–Entity* edges from the knowledge graph. It turns out the error is 4.3% higher without using the results of RE. As a result, our information extraction modules can significantly improve the performance of pandemic forecasts.

#### (ii) Pandemic trend prediction

Since risk factor prediction is essentially time-series prediction tasks, we follow a similar evaluation routine to evaluate our forecasting algorithm. We apply the Mean Absolute Error (MAE) to evaluate the short-term (1,7 days ahead) and long-term (14,28 days ahead) pandemic forecast performances on both confirmed cases and fatality numbers.

We collect the results of the compartment models from the COVID-19 Forecast Hub.^6^ Note that the 1-day-ahead results of the compartment models are not provided in the COVID-19 Forecast Hub. We implement all other baselines and achieve the results to compare with our method. As shown in table 1, our model outperforms the state-of-the-art baselines, MPNN+LSTM, on both confirmed case and fatality forecasts. We notice that our model initially outperforms the baselines by a small margin (1-day-ahead forecast) while the improvement becomes more significant as the prediction time span grows larger. While LSTM achieves satisfactory performance when we use it to predict the 28-day-ahead fatality numbers, the errors are growing rapidly when we use it to conduct the short-term forecasts. We believe LSTM, as a conventional sequence modelling method, is incapable of tackling the sequential inputs with sharp changes. The MAE scores of the compartment models, such as JHU_IDD-CovidSP, are also exploding in confirmed case prediction. We surmise that the compartment models always assume the peak of the pandemic comes after the current data and handle the predictions in the early stage poorly.

**Table 1.** Performance of the short-term (1 day and 7 days ahead) and long-term (14 days and 28 days ahead) new confirmed case number and fatality forecasts. All the improvements of our method over the baseline methods are statistically significant at a 99% confidence level in paired t-tests. Our method achieves 5.6%, 9.5%, 9.4% and 5.6% lower MAE than the best baseline MPNN + LSTM when forecasting the new confirmed case numbers for 1, 7, 14, 28 days ahead.

	confirmed case				fatality			
no. days ahead (r)	1	7	14	28	1	7	14	28
RobertWalraven-ESG	—	768.43	978.53	2472.09	—	15.49	18.59	26.18
UCLA-SuEIR	—	755.36	1099.76	1591.01	—	14.24	15.60	19.06
JHU_IDD-CovidSP	—	1123.72	1253.14	1534.64	—	18.91	19.85	24.36
ARIMA	604.18	802.98	961.30	1300.49	19.32	21.91	24.47	29.20
PROPHET	791.07	991.05	1341.80	2019.24	16.59	18.65	22.22	31.77
LSTM	1262.33	1248.08	1235.20	1204.19	18.04	17.94	17.77	17.74
MPNN	485.52	567.74	825.41	1304.11	12.13	12.90	14.87	19.73
MPNN+LSTM	455.68	523.77	672.05	967.12	12.17	12.79	14.57	20.01
ours	430.01	474.16	608.98	913.20	11.78	11.85	13.24	18.26

## 5. Conclusion

In this paper, we present a novel pandemic surveillance system based on social media data, COVID-19 Surveiller, with two parts, including social media mining and robust full-stack web system development. In social media mining, we construct a dynamic knowledge graph by named entity recognition and RE. Based on the derived dynamic knowledge graph, we learn a dynamic graph neural network to predict the pandemic trends, such as case numbers and fatalities. The robust full-stack web system further uses the predictions from social media mining to present a user-friendly interface for users to monitor the pandemics across different areas. We also conduct sufficient experiments to demonstrate that our social media mining model can accurately predict the pandemic trends and outperform state-of-the-art methods. We also show some examples to present how our web system can help users more conveniently monitor the pandemics.

## Footnotes

1 https://coronavirus.jhu.edu/map.html.

2 www.worldometers.info/coronavirus/.

3 https://coronavirus.1point3acres.com/.

4 https://developer.twitter.com/en/docs/labs/covid19-stream/api-reference/get-tweets-stream-covid19.

5 www.safegraph.com/.

6 https://github.com/reichlab/covid19-forecast-hub.

## Data accessibility

We use data from Twitter, Safegraph, as well as public data provided by C.D.C. Twitter and Safegraph require users to register and sign agreement before accessing their data.
